# Association Between Cushing’s Syndrome and Sleep Apnea: Results From the National Inpatient Sample

**DOI:** 10.7759/cureus.22044

**Published:** 2022-02-09

**Authors:** Meghana Pattipati, Goutham Gudavalli

**Affiliations:** 1 Internal Medicine, The Brooklyn Hospital Center, Brooklyn, USA; 2 Critical Care Medicine, Rapides Regional Medical Center, Alexandria, USA

**Keywords:** cushing’s syndrome, obesity, central sleep apnea, sleep apnea, osa

## Abstract

Background

Cushing’s syndrome is a metabolic disorder related to excess cortisol production. Patients with Cushing’s syndrome are at risk for the development of other comorbid medical conditions such as hypertension, diabetes, obesity, and obstructive sleep apnea. Obstructive sleep apnea has been well associated with endocrine disorders such as acromegaly and hypothyroidism. However, its causal association with Cushing’s syndrome is still unclear. We utilized a national database to study the prevalence of sleep apnea in Cushing’s syndrome.

Hypothesis

We hypothesized that patients with Cushing’s syndrome might have an increased prevalence of sleep apnea.

Methods

Patients aged above 18 years from the NIS database between 2017 and 2018 with a diagnosis of Cushing’s syndrome and sleep apnea were extracted using the 10th revision of the International Classification of Diseases (ICD-10) codes, with code E24 representing Cushing’s syndrome and G47.3 representing sleep apnea. The prevalence of sleep apnea and other comorbid medical conditions were identified using the ICD-10 codes. Logistic regression analysis was performed to examine the association between Cushing’s syndrome and sleep apnea.

Results

Cushing’s syndrome was prevalent in 0.037% (2,248 of 6,023,852) of all inpatient hospitalizations. Patients with Cushing’s syndrome were slightly younger (mean age: 54 ± 16 versus 58 ± 20) and more likely to be females (76%, 1,715 out of 2,248) and had higher rates of sleep apnea (21.9% versus 8.7%, p < 0.000) and obstructive sleep apnea (OSA) (18.6% versus 7.2%, p < 0.000) when compared to the general population. Cushing’s syndrome is independently associated with sleep apnea, with an unadjusted odds ratio (OR) of 2.94 (p < 0.01) and an adjusted odds ratio (aOR) of 1.79 after adjusting for demographics and other risk factors for sleep apnea and comorbid medical conditions (p < 0.01).

Conclusions

Cushing’s syndrome is associated with increased prevalence of sleep apnea and independent predictor of sleep apnea. Further prospective studies are recommended to validate the causal association. The high prevalence and coexistence of both these disorders validate screening for sleep apnea as part of routine workup in patients with Cushing’s syndrome and vice versa.

## Introduction

Cushing’s syndrome is a metabolic disorder related to excess cortisol production. Patients with Cushing’s syndrome are at risk for the development of other comorbid medical conditions such as hypertension, diabetes, and obesity. Apnea is defined by the American Academy of Sleep Medicine (AASM) as the cessation of airflow for at least 10 seconds. Obstructive sleep apnea (OSA) and central sleep apnea are included under the umbrella, with OSA being the most common type of sleep apnea. Obstructive sleep apnea has been well associated with endocrine disorders such as acromegaly and hypothyroidism. However, its causal association with Cushing’s syndrome is still unclear. There are several different prospects regarding the development of sleep apnea in patients with Cushing’s syndrome, one of which could be secondary to impaired cortisol release. It has been studied in the literature that there is some association between sleep and cortisol secretion. The exact mechanism of the prevalence or coexistence of sleep apnea, both central sleep apnea and obstructive sleep apnea, has not been well described. There are studies that found a relationship between Cushing’s syndrome and OSA. The correlation between obesity and fat tissue accumulation in the neck likely leads to the development of OSA in patients with Cushing’s syndrome/disease [1]. However, our study aimed to investigate if Cushing’s syndrome is an independent predictor of sleep apnea after adjusting for obesity and other comorbidities predisposing to sleep apnea. Parapharyngeal fat accumulation in Cushing’s syndrome/disease can cause sleep apnea, but no epidemiological information is available [2]. The very first association between OSA and Cushing’s syndrome was reported by Shipley et al. in 1992; 32% had mild sleep apnea (apnea-hypopnea index (AHI) > 9.4 events/hour), and 18% had ≥17.5 events/hour [3]. A nationwide longitudinal study done in Taiwan in 2017 investigated 1,612 patients with Cushing’s syndrome, and it showed a 2.82-fold increased risk of developing obstructive sleep apnea [4]. This study encourages further research into this association, as the mechanisms underlying this phenomenon remain unclear. Also, this study only included the incidence of OSA, but not sleep apnea in general, which included central sleep apnea and OSA [4]. A study conducted on women with Cushing’s syndrome found that women with Cushing’s syndrome are two times more likely to have obstructive sleep apnea, and cortisol was found to be an independent predictor of apnea-hypopnea index (AHI) after controlling for BMI and homeostasis model assessment (HOMA) score and plays a major role in the pathogenesis of OSA [5]. In a study conducted by Berger et al., three-month exogenous steroid therapy on an objective measure of sleep-disordered breathing showed that one out of 17 patients increased their mean AHI by 56%; however, the body weight, neck girth changes, and cumulative steroid doses were not correlated to the AHI increment [6]. Sleep apnea in Cushing’s syndrome could be secondary to impaired cortisol release. A study has shown the effect of the levels of serum cortisol on various stages of sleep, where REM sleep was found to be present when cortisol concentrations were decreasing, and wakefulness and stage 1 sleep are associated with increased cortisol concentrations [6].

Our research aims to address the question of whether sleep apnea should be considered independent comorbidity of Cushing’s syndrome and should screening for OSA be part of the routine workup for patients with Cushing’s syndrome. OSA comorbidity in Cushing’s syndrome can be a risk factor for increased morbidity and mortality and might have a major effect on the quality of life.

## Materials and methods

Data source

We utilized the AHRQ’s NIS database, which is developed as part of the Healthcare Cost and Utilization Project (HCUP). The NIS is the largest all-payer inpatient healthcare database in the United States. It included data from approximately seven million patient hospital stays per year from over 1,000 hospitals and is a representative sample of about 20% of nonfederal hospitals in the United States.

Patient population

Data capture in the NIS databases from 2017 to 2018 was utilized for this analysis. All patients above 18 years of age with a diagnosis of Cushing’s syndrome were identified using the Clinical Classification Software (CCS) codes. The CCS is a categorization scheme that groups the 10th revision of the International Classification of Diseases (ICD-10) codes into mutually exclusive categories. CCS code E24 represents all ICD-10 diagnoses of Cushing’s syndrome. The sleep apnea group included all kinds of sleep apnea, including central and obstructive sleep apnea, and other causes representing the ICD-10 diagnostic code of G47.3, whereas obstructive sleep apnea is separately represented by code OSA G47.33. All the above diagnostic codes were obtained if it was included in the 40 diagnostic codes listed in the database.

The baseline demographics and social variables examined in the study included age, gender, race, smoking, alcohol, various comorbidities (hypertension, diabetes, obesity, liver disease, chronic lung disease, chronic kidney disease/end-stage renal disease, cerebral infarction, heart failure, cardiac arrhythmias, thyroid disorder, obesity hypoventilation syndrome (OHS), restless leg syndrome (RLS), and fluid, electrolyte, and acid-base disorders), social factors such as insurance payer, hospital bed size, socioeconomic status based on household income, location and region of the hospital, and teaching status of the hospital.

Statistical analysis

The primary outcome of the study is to estimate the prevalence of sleep apnea and OSA in patients diagnosed with Cushing’s syndrome and predict the independent association after adjusting for other parameters such as obesity, substance use (smoking and alcohol), and underlying comorbidities. All analyses were performed using STRATA/SE 17.0. Univariate analysis was performed initially to estimate the individual risk factors and predictors for Cushing’s syndrome using logistic regression for numerical covariates and weighted chi-square tests for categorical covariates. Multivariate logistic regression analysis was performed based on the univariate analysis to predict the adjusted odds ratio (aOR) for each variable of interest.

## Results

Cushing’s syndrome was prevalent in 2,248 of 6,023,852 hospitalized patient samples, estimating the prevalence to be 0.037%. Sleep apnea was diagnosed in 493 patients among the 2,248 (21.9%) compared with 525,079 among the 6,021,604 (8.7%) of the general population (control). Table 1 lists the baseline demographics of patients in both groups of patients with and without Cushing’s syndrome. Significant differences were found between both groups, with patients in the Cushing’s syndrome group being slightly younger compared to the general population (mean age ± SD: 54 ± 16 versus 58 ± 20, p < 0.01). The majority of them were females (76% versus 57%), and Cushing’s syndrome is more prevalent in the White population (73% versus 67%). The prevalence of certain comorbidities was higher in the Cushing’s syndrome group versus the general population (diabetes: 47.5% versus 27.5%, obesity: 41% versus 16.8%, hypertension: 69.7% versus 56.6%, chronic lung disease: 35.6% versus 22.6%, chronic kidney disease/end-stage renal disease: 19.4% versus 17.2%, chronic liver disease: 7.7% versus 5.5%, heart failure: 24% versus 17.7%, sleep apnea: 21.9% versus 8.7%, OSA: 18.6% versus 7.2%, OHS: 3.6% versus 0.6%, thyroid disorders: 24.6% versus 13.4%).

Patients in the Cushing’s syndrome group have fallen into the higher socioeconomic status category in terms of higher income compared with the control (less than 50th percentile group: 57% versus 52%, with major difference noted in the private insurance group, 33.4% in the Cushing’s syndrome group versus 26.3% among others).

**Table 1:** Baseline demographics of the general population with and without Cushing’s syndrome

	Control (general population) (N = 6,021,604)	Control (general population) (% = 99.96%)	Cushing’s syndrome (N = 2,248)	Cushing’s syndrome (% = 0.037%)	P value
Sex: Male	Reference				
Female	3,450,511	57%	1,715	76%	<0.001
Age in years					
18–19	43,436	0.7%	18	0.8%	<0.001
≥20 and <30	651,487	10.8%	167	7.4%	<0.001
≥30 and <40	715,922	11.9%	287	12.7%	<0.001
≥40 and <50	541,640	9%	358	15.9%	<0.001
≥50 and <60	870,878	14.5%	513	22.8%	0.144
≥60 and <70	1,128,762	18.7%	507	22.5%	<0.001
≥70 and <80	1,073,623	17.8%	294	13%	<0.001
≥80 and <90	747,084	12.4%	96	4.3%	<0.001
≥90 and <100	248,500	4.1%	8	0.35%	<0.001
Mean age in years at admission (mean ± SD)	58 ± 20		54 ± 16		
Diabetes	1,653,951	27.5%	1,069	47.5%	<0.001
Obesity	1,013,497	16.8%	923	41%	<0.001
Hypertension	3,410,907	56.6%	1,567	69.7%	<0.001
Chronic lung disease	1,361,787	22.6%	801	35.6%	<0.001
Chronic kidney disease/ESRD	1,035,714	17.2%	436	19.4%	<0.008
Liver disease	329,552	5.5%	173	7.7%	<0.09
Cerebral infarction	134,560	2.2%	47	2.1%	<0.304
Heart failure	1,066,315	17.7%	540	24%	<0.001
Sleep apnea	525,079	8.7%	493	21.9%	<0.001
Obstructive sleep apnea	433,303	7.2%	418	18.6%	<0.542
Obesity hypoventilation syndrome	36,554	0.6%	81	3.6%	<0.001
Thyroid disorders	809,272	13.4%	554	24.6%	<0.001
Fluid, electrolyte, and acid–base abnormalities	1,510,937	25%	831	37%	<0.001
Atrial fibrillation/flutter	948,272	15.7%	293	13%	<0.026
Other cardiac arrhythmias	110,723	1.8%	36	1.6%	<0.423
RLS	74,719	1.24%	48	2.1%	<0.001
Smoking	1,009,738	16.7%	270	12%	<0.001
Household income (percentile)					
0–25th	1,753,964	30%	541	24.3%	<0.001
26th–50th	1,599,032	27%	615	27.7%	<0.001
51st–75th	1,406,404	23.7%	582	26.2%	<0.001
76th–100th	1,154,711	19.5%	480	21.6%	<0.001
Race					
White	3,933,806	67%	1,608	73%	<0.001
Black	884,709	15%	244	11%	<0.001
Hispanic	673,605	11.5%	212	9.6%	<0.001
Asian/Pacific Island	163,754	2.8%	60	2.7%	<0.271
Native American	38,039	0.6%	14	0.6%	<0.293
Other	177,439	3%	60	2.7%	<0.144
Pay					
Medicare	2,902,371	48.3%	1,035	46%	<0.001
Medicaid	1,089,273	18%	340	15%	<0.001
Private	1,586,714	26.3%	751	33.4%	<0.039
Self-pay	247,165	4.1%	71	3.2%	<0.001
No charge	21,637	0.35%	1	0.04%	<0.038
Other	166,460	2.76%	47	2%	<0.015
Location of hospital/teaching status					
Rural	537,907	8.9%	160	7.1%	<0.001
Urban non-teaching	1,252,816	20.8%	375	16.7%	<0.747
Urban teaching	4,230,881	70%	1,713	76.2%	<0.001
Bed size					
Small	1,277,522	21%	387	17.2%	<0.001
Medium	1,758,086	29%	601	26.7%	<0.107
Large	2,985,996	49.6%	1,260	56%	<0.001
Hospital region					
Northeast	118,422	2%	461	20.5%	<0.001
Midwest	1,345,585	22%	537	23.8%	<0.004
South	2,382,075	39.5%	815	36.2%	<0.002
West	1,175,522	19.5%	435	19.3%	<0.052
Died during hospitalization	135,773	2.25%	80	3.56%	<0.001

Table 2 describes the baseline patient characteristics in the patient population with and without sleep apnea. Sleep apnea was more prevalent in Whites (75.7% versus 66%) and in patients who are slightly older than the general population (mean age ± SD: 64 ± 13 versus 57 ± 20). Unlike Cushing’s syndrome, sleep apnea is more prevalent in males than in females, with female cases accounting for 43% versus 58.6% in the general population. The comorbidities that are more prevalent in the sleep apnea group compared with the control group were diabetes (48% versus 25.5%), obesity (48.4% versus 13.8%), hypertension (81.6% versus 54.3%), Cushing’s syndrome (0.09% versus 0.03%), chronic lung disease (40% versus 21%), chronic kidney disease/ESRD (27.6% versus 16.2%), chronic liver disease (6.4% versus 5.4%), heart failure (34% versus 16%), atrial fibrillation (27.8% versus 14.6%), other cardiac arrhythmias (2.8% versus 1.74%), obesity hypoventilation syndrome (1.35% versus 0.5%), thyroid disorders (18.6% versus 13%), and restless leg syndrome (3.7% versus 1%).

The comorbidities that are less prevalent in sleep apnea patients compared with the control group were cerebral infarction (2% versus 2.25%), smoking (13.5% versus 17%), and alcohol-related disorders (3.4% versus 6.3%). Patients in the sleep apnea group were relatively under the low socioeconomic group with Medicare, Medicaid, and self-pay being the primary insurance type (78.4% versus 70.3%).

**Table 2:** Baseline demographics of patients with and without sleep apnea

	General population without sleep apnea (N = 5,498,280)	General population without sleep apnea (% = 91.28%)	Sleep apnea (N = 525,572)	Sleep apnea (% = 8.72%)	P value
Sex					
Male	2,273,084	41.3%	297,995	56.7%	<0.001
Female	3,224,674	58.6%	227,552	43.3%	<0.001
Age in years					
18–19	43,070	0.78%	384	0.07%	<0.001
≥20 and <30	645,291	11.7%	6,363	1.2%	0.489
≥30 and <40	696,535	12.7%	19,674	3.7%	<0.001
≥40 and <50	493,839	9%	48,159	9.2%	<0.001
≥50 and <60	767,841	14%	103,550	19.7%	<0.001
≥60 and <70	978,539	17.8%	150,730	28.7%	<0.001
≥70 and <80	940,113	17%	133,804	25.4%	<0.001
≥80 and <90	691,539	12.6%	55,641	10.6%	0.606
≥90 and <100	241,249	4.4%	7,259	1.4%	<0.001
Mean age in years at admission (mean ± SD)	57±20		64±13		
Cushing’s syndrome	1,755	0.03%	493	0.09%	<0.001
Diabetes	1,402,449	25.5%	252,571	48%	<0.001
Obesity	760,175	13.8%	254,245	48.4%	<0.001
Hypertension	2,983,757	54.3%	428,717	81.6%	<0.001
Chronic lung disease	1,151,029	21%	211,559	40%	<0.001
Chronic kidney disease/ESRD	891,063	16.2%	145,087	27.6%	<0.001
Liver disease	295,925	5.4%	33,800	6.4%	<0.001
Cerebral infarction	124,201	2.25%	10,406	2%	<0.001
Heart failure	887,719	16%	179,136	34%	<0.001
Obesity hypoventilation syndrome	29,510	0.5%	7,125	1.35%	<0.001
Thyroid disorders	711,727	13%	98,099	18.6%	<0.001
Fluid, electrolyte, and acid–base disorders	1,373,143	25%	138,625	26%	<0.001
Atrial fibrillation/flutter	802,520	14.6%	146,045	27.8%	<0.001
Other cardiac arrhythmias	95,881	1.74%	14,878	2.8%	<0.001
RLS	55,020	1%	19,747	3.7%	<0.001
Smoking	939,200	17%	70,808	13.5%	<0.001
Alcohol-related disorders	346,704	6.3%	17,930	3.4%	<0.001
Household income (percentile)					
0–25th	1,612,954	29.9%	141,551	27.3%	<0.001
26th–50th	1,455,384	27%	144,263	27.8%	<0.001
51st–75th	1,275,260	23.6%	131,726	25.4%	<0.001
76th–100th	1,054,593	19.5%	100,598	19.4%	<0.001
Race					
White	3,547,515	66%	387,899	75.7%	<0.001
Black	812,434	15%	72,519	14%	<0.001
Hispanic	639,660	12%	34,157	6.7%	<0.001
Asian/Pacific Island	157,786	3%	6,028	1.2%	<0.001
Native American	35,345	0.65%	2,708	0.5%	<0.001
Other	168,548	3.14%	8,951	1.75%	<0.001
Pay					
Medicare	2,581,830	47%	321,576	61.2%	<0.001
Medicaid	1,039,816	19%	49,797	9.5%	<0.001
Private	1,457,309	26.5%	130,156	24.8%	0.763
Self-pay	238,318	4.3%	8,918	1.7%	<0.001
No charge	20,971	0.4%	667	0.13%	<0.001
Other	152,653	2.8%	13,854	2.6%	<0.001
Location of hospital/teaching status					
Rural	495,305	9%	42,762	8.1%	<0.001
Urban non-teaching	1,148,887	20.9%	104,304	19.8%	<0.001
Urban teaching	3,854,088	70.1%	378,506	72%	<0.001
Bed size					
Small	1,167,980	21.2%	109,929	21%	<0.001
Medium	1,608,347	29.2%	150,340	28.6%	<0.001
Large	2,721,953	49.5%	265,303	50.5%	<0.001
Hospital region					
Northeast	1,033,267	18.8%	85,616	16.3%	<0.001
Midwest	1,192,925	21.7%	153,197	20.1%	<0.001
South	2,189,730	40%	193,160	36.7%	<0.001
West	1,082,358	19.7%	93,599	17.8%	<0.001

Independent variables associated with sleep apnea	Unadjusted odds ratio (CI)	P value	Adjusted odds ratio (CI)	P value
Sex				
Male	Reference			
Female	0.53 (0.53–0.54)	<0.001	0.55 (0.54–0.55)	<0.001
Age in years				
18–19	Reference			
≥20 and <30	1.10 (0.99–1.22)	0.056	1.03 (0.93–1.15)	0.589
≥30 and <40	3.16 (2.86–3.50)	<0.001	2.23 (2.01–2.48)	<0.001
≥40 and <50	10.9 (9.88–12.09)	<0.001	4.54 (4.09–5.04)	<0.001
≥50 and <60	15.12 (13.7–16.72)	<0.001	4.82 (4.34–5.34)	<0.001
≥60 and <70	17.27 (15.7–19.10)	<0.001	4.32 (3.89–4.79)	<0.001
≥70 and <80	15.96 (14.43–17.7)	<0.001	3.52 (3.17–3.91)	<0.001
≥80 and <90	9.02 (8.15–9.98)	<0.001	2.15 (1.94–2.39)	<0.001
≥90 and <100	3.37 (3.04–3.74)	<0.001	0.97 (0.87–1.08)	0.606
Cushing’s syndrome				
No	Reference			
Yes	2.94 (2.66–3.24)	<0.001	1.79 (1.60–2.01)	<0.001
Diabetes				
No	Reference			
Yes	2.70 (2.68–2.71)	<0.001	1.38 (1.37–1.39)	<0.001
Obesity				
No	Reference			
Yes	5.84 (5.80–5.97)	<0.001	4.59 (4.56–4.62)	<0.001
Hypertension				
No	Reference			
Yes	3.73 (3.70–3.75)	<0.001	1.70 (1.68–1.71)	<0.001
Chronic lung disease				
No	Reference			
Yes	2.54 (2.52–2.55)	<0.001	1.96 (1.94–1.97)	<0.001
Chronic kidney disease/ESRD				
No	Reference			
Yes	1.97 (1.95–1.98)	<0.001	1.05 (1.04–1.06)	<0.001
Liver disease				
No	Reference			
Yes	1.20 (1.19–1.22)	<0.001	1.05 (1.04–1.07)	<0.001
Cerebral infarction				
No	Reference			
Yes	0.87 (0.85–0.89)	<0.001	0.77 (0.75–0.78)	<0.001
Heart failure				
No	Reference			
Yes	2.68 (2.66–2.70)	<0.001	1.43 (1.42–1.44)	<0.001
Obesity hypoventilation syndrome				
No	Reference			
Yes	2.54 (2.48–2.61)	<0.001	0.39 (0.38–0.40)	<0.001
Thyroid disorders				
No	Reference			
Yes	1.54 (1.53–1.55)	<0.001	1.28 (1.27–1.29)	<0.001
Fluid, electrolyte, and acid–base disorders				
No	Reference			
Yes	1.07 (1.06–1.08)	<0.001	0.80 (0.79–0.80)	<0.001
Atrial fibrillation/flutter				
No	Reference			
Yes	2.25 (2.23–2.26)	<0.001	1.42 (1.41–1.43)	<0.001
Other cardiac arrhythmias				
No	Reference			
Yes	1.64 (1.61–1.67)	<0.001	1.14 (1.12–1.16)	<0.001
RLS				
No	Reference			
Yes	3.86 (3.79–3.92)	<0.001	2.68 (2.63–2.73)	<0.001
Smoking				
No	Reference			
Yes	0.75 (0.74–0.76)	<0.001	0.69 (0.69–0.70)	<0.001
Alcohol use				
No	Reference			
Yes	0.52 (0.51–0.53)	<0.001	0.61 (0.60–0.62)	<0.001
Household income (percentile)				
0–25th	Reference			
26th–50th	1.12 (1.12–1.13)	<0.001	1.10 (1.09–1.11)	<0.001
51st–75th	1.17 (1.16–1.18)	<0.001	1.18 (1.17–1.19)	<0.001
76th–100th	1.08 (1.07–1.09)	<0.001	1.24 (1.22–1.25)	<0.001
Race				
White	Reference			
Black	0.81 (0.80–0.82)	<0.001	0.86 (0.85–0.87)	<0.001
Hispanic	0.48 (0.482–0.493)	<0.001	0.64 (0.63–0.64)	<0.001
Asian/Pacific Island	0.34 (0.340–0.358)	<0.001	0.50 (0.49–0.51)	<0.001
Native American	0.70 (0.67–0.72)	<0.001	0.80 (0.76–0.83)	<0.001
Other	0.48 (0.47–0.49)	<0.001	0.64 (0.62–0.65)	<0.001
Pay				
Medicare	Reference			
Medicaid	0.384 (0.38–0.388)	<0.001	0.69 (0.68–0.70)	<0.001
Private	0.71 (0.71–0.72)	<0.001	0.99 (0.98–1.00)	0.763
Self-pay	0.30 (0.29–0.30)	<0.001	0.52 (0.51–0.53)	<0.001
No charge	0.25 (0.23–0.27)	<0.001	0.43 (0.39–0.47)	<0.001
Other	0.72 (0.71–0.74)	<0.001	0.96 (0.94–0.98)	<0.001
Location of hospital/teaching status				
Rural	Reference			
Urban non-teaching	1.05 (1.03–1.06)	<0.001	1.05 (1.03–1.06)	<0.001
Urban teaching	1.13 (1.12–1.14)	<0.001	1.20 (1.18–1.21)	<0.001
Bed size				
Small	Reference			
Medium	0.99 (0.98–1.00)	<0.001	1.03 (1.02–1.04)	<0.001
Large	1.03 (1.02–1.04)	<0.001	1.06 (1.06–1.07)	<0.001
Hospital region				
Northeast	Reference			
Midwest	1.54 (1.53–1.56)	<0.001	1.40 (1.39–1.42)	<0.001
South	1.06 (1.05–1.07)	<0.001	1.10 (1.09–1.11)	<0.001
West	1.04 (1.05–1.06)	<0.001	1.23 (1.22–1.24)	<0.001

Table 3 describes the odds ratio (OR) and the adjusted odds ratio (aOR) of sleep apnea and the variables of interest. The odds of exposure to certain risk factors were calculated for sleep apnea, and the results showed that sleep apnea is independently associated with the following conditions. Cushing’s syndrome is found to have an independent association with sleep apnea, with an unadjusted odds ratio of 2.94 and an adjusted odds ratio of 1.79 after adjusting for multiple risk factors. Obesity had the strongest association with sleep apnea (OR = 5.84, 95%CI = 5.80-5.97; aOR = 4.59, 95%CI = 4.56-4.62), followed by chronic lung disease (OR = 2.54, 95%CI = 2.52-2.55; aOR = 1.96, 95%CI = 1.94-1.97), hypertension (OR = 3.73, 95%CI = 3.70-3.75; aOR = 1.70, 95%CI = 1.68-1.71), restless leg syndrome (OR = 3.86, 95%CI = 3.79-3.92; aOR = 1.70, 95%CI = 1.68-1.71), diabetes (OR = 2.70, 95%CI = 2.68-2.71; aOR = 1.38, 95%CI = 1.37 1.39), heart failure (OR = 2.68, 95%CI = 2.66-2.70; aOR = 1.43, 95%CI = 1.42-1.44), atrial fibrillation/atrial flutter (OR = 2.25, 95%CI = 2.23-2.26; aOR = 1.42, 95%CI = 1.41-1.43), other cardiac arrhythmias (OR = 1.64, 95%CI = 1.61-1.67; aOR = 1.14, 95%CI = 1.12-1.16), thyroid disorders (OR = 1.54, 95%CI = 1.53-1.55; aOR = 1.28, 95%CI = 1.27-1.29), chronic kidney disease/ESRD (OR = 1.97, 95%CI = 1.95-1.98; aOR = 1.05, 95%CI = 1.04-1.06), and chronic liver disease (OR = 1.20, 95%CI = 1.19-1.22; aOR = 1.05, 95%CI = 1.04-1.07). Univariate and multivariate analyses were performed for the statistical significance of these conditions.

**Table 3:** Adjusted odds ratio for each independent variable associated with sleep apnea

Independent variables associated with sleep apnea	Unadjusted odds ratio (CI)	P value	Adjusted odds ratio (CI)	P value
Sex				
Male	Reference			
Female	0.53 (0.53–0.54)	<0.001	0.55 (0.54–0.55)	<0.001
Age in years				
18–19	Reference			
≥20 and <30	1.10 (0.99–1.22)	0.056	1.03 (0.93–1.15)	0.589
≥30 and <40	3.16 (2.86–3.50)	<0.001	2.23 (2.01–2.48)	<0.001
≥40 and <50	10.9 (9.88–12.09)	<0.001	4.54 (4.09–5.04)	<0.001
≥50 and <60	15.12 (13.7–16.72)	<0.001	4.82 (4.34–5.34)	<0.001
≥60 and <70	17.27 (15.7–19.10)	<0.001	4.32 (3.89–4.79)	<0.001
≥70 and <80	15.96 (14.43–17.7)	<0.001	3.52 (3.17–3.91)	<0.001
≥80 and <90	9.02 (8.15–9.98)	<0.001	2.15 (1.94–2.39)	<0.001
≥90 and <100	3.37 (3.04–3.74)	<0.001	0.97 (0.87–1.08)	0.606
Cushing’s syndrome				
No	Reference			
Yes	2.94 (2.66–3.24)	<0.001	1.79 (1.60–2.01)	<0.001
Diabetes				
No	Reference			
Yes	2.70 (2.68–2.71)	<0.001	1.38 (1.37–1.39)	<0.001
Obesity				
No	Reference			
Yes	5.84 (5.80–5.97)	<0.001	4.59 (4.56–4.62)	<0.001
Hypertension				
No	Reference			
Yes	3.73 (3.70–3.75)	<0.001	1.70 (1.68–1.71)	<0.001
Chronic lung disease				
No	Reference			
Yes	2.54 (2.52–2.55)	<0.001	1.96 (1.94–1.97)	<0.001
Chronic kidney disease/ESRD				
No	Reference			
Yes	1.97 (1.95–1.98)	<0.001	1.05 (1.04–1.06)	<0.001
Liver disease				
No	Reference			
Yes	1.20 (1.19–1.22)	<0.001	1.05 (1.04–1.07)	<0.001
Cerebral infarction				
No	Reference			
Yes	0.87 (0.85–0.89)	<0.001	0.77 (0.75–0.78)	<0.001
Heart failure				
No	Reference			
Yes	2.68 (2.66–2.70)	<0.001	1.43 (1.42–1.44)	<0.001
Obesity hypoventilation syndrome				
No	Reference			
Yes	2.54 (2.48–2.61)	<0.001	0.39 (0.38–0.40)	<0.001
Thyroid disorders				
No	Reference			
Yes	1.54 (1.53–1.55)	<0.001	1.28 (1.27–1.29)	<0.001
Fluid, electrolyte, and acid–base disorders				
No	Reference			
Yes	1.07 (1.06–1.08)	<0.001	0.80 (0.79–0.80)	<0.001
Atrial fibrillation/flutter				
No	Reference			
Yes	2.25 (2.23–2.26)	<0.001	1.42 (1.41–1.43)	<0.001
Other cardiac arrhythmias				
No	Reference			
Yes	1.64 (1.61–1.67)	<0.001	1.14 (1.12–1.16)	<0.001
RLS				
No	Reference			
Yes	3.86 (3.79–3.92)	<0.001	2.68 (2.63–2.73)	<0.001
Smoking				
No	Reference			
Yes	0.75 (0.74–0.76)	<0.001	0.69 (0.69–0.70)	<0.001
Alcohol use				
No	Reference			
Yes	0.52 (0.51–0.53)	<0.001	0.61 (0.60–0.62)	<0.001
Household income (percentile)				
0–25th	Reference			
26th–50th	1.12 (1.12–1.13)	<0.001	1.10 (1.09–1.11)	<0.001
51st–75th	1.17 (1.16–1.18)	<0.001	1.18 (1.17–1.19)	<0.001
76th–100th	1.08 (1.07–1.09)	<0.001	1.24 (1.22–1.25)	<0.001
Race				
White	Reference			
Black	0.81 (0.80–0.82)	<0.001	0.86 (0.85–0.87)	<0.001
Hispanic	0.48 (0.482–0.493)	<0.001	0.64 (0.63–0.64)	<0.001
Asian/Pacific Island	0.34 (0.340–0.358)	<0.001	0.50 (0.49–0.51)	<0.001
Native American	0.70 (0.67–0.72)	<0.001	0.80 (0.76–0.83)	<0.001
Other	0.48 (0.47–0.49)	<0.001	0.64 (0.62–0.65)	<0.001
Pay				
Medicare	Reference			
Medicaid	0.384 (0.38–0.388)	<0.001	0.69 (0.68–0.70)	<0.001
Private	0.71 (0.71–0.72)	<0.001	0.99 (0.98–1.00)	0.763
Self-pay	0.30 (0.29–0.30)	<0.001	0.52 (0.51–0.53)	<0.001
No charge	0.25 (0.23–0.27)	<0.001	0.43 (0.39–0.47)	<0.001
Other	0.72 (0.71–0.74	<0.001	0.96 (0.94–0.98)	<0.001
Location of hospital/teaching status				
Rural	Reference			
Urban non-teaching	1.05 (1.03–1.06)	<0.001	1.05 (1.03–1.06)	<0.001
Urban teaching	1.13 (1.12–1.14)	<0.001	1.20 (1.18–1.21)	<0.001
Bed size				
Small	Reference			
Medium	0.99 (0.98–1.00)	<0.001	1.03 (1.02–1.04)	<0.001
Large	1.03 (1.02–1.04)	<0.001	1.06 (1.06–1.07)	<0.001
Hospital region				
Northeast	Reference			
Midwest	1.54 (1.53–1.56)	<0.001	1.40 (1.39–1.42)	<0.001
South	1.06 (1.05–1.07)	<0.001	1.10 (1.09–1.11)	<0.001
West	1.04 (1.05–1.06)	<0.001	1.23 (1.22–1.24)	<0.001

## Discussion

Cushing’s syndrome is an independent risk factor for the development of sleep apnea. Oftentimes, both conditions are coexistent, and the burden of unrecognized and untreated sleep apnea on health-related quality of life is well known. Sleep apnea still remains an underdiagnosed medical condition, and this study reinforces the basic necessity to screen for sleep apnea during routine clinical practice in high-risk patients, including those with Cushing’s syndrome. The morbidity and mortality of untreated sleep apnea are well known and could have a slightly higher effect in subpopulation groups such as those with Cushing’s syndrome.

In a meta-analysis of 637 participants with OSA, CPAP treatment significantly reduced both plasma and salivary cortisol levels. Individuals undergoing investigation for Cushing’s syndrome would benefit from an initial screening for OSA; the impact of CPAP on cortisol has been debatable because of conflicting findings between studies due to small sample sizes [7]. The mechanism of correlation between sleep apnea and Cushing’s disease/Cushing’s syndrome have never been investigated; it has been suggested that weight gain and adipose tissue accumulation according to a centripetal pattern in the subcutaneous tissue of the neck can likely lead to the development of obstructive sleep apnea in these patient population. The neck and waist circumference are highly predictive of OSA severity [8,9].

Our study is the largest to date to evaluate patients with Cushing’s syndrome and sleep apnea in the United States. The underlying pathophysiology of the link between these two disease processes is yet to be determined, and further prospective studies have to be conducted to study the exact pathophysiology of the association. This is a large sample study with statistically significant results. Despite the large power afforded by the large number of patients available in NIS, there are several significant limitations of this study; given that this database is based on administrative coding, not all clinical data are available for analysis. For this reason, it is not possible to definitely identify if patients with sleep apnea developed Cushing’s syndrome or patients with Cushing’s syndrome developed sleep apnea later. Also, the treatment options and apnea-hypopnea index (AHI) determining the severity of sleep apnea were also not included. Most of the patients with sleep apnea or Cushing’s syndrome without any underlying comorbidities might not have been hospitalized, which underpredicts the overall prevalence. Our findings however highlight the need for further prospective studies to clarify the coexistence of these two disorders and the need for incorporating routine screening for either condition in patients diagnosed with one of those to improve the outcomes in these patient populations. Another limitation of our analysis is that the NIS does not capture individual treatment data, and thus, we are unable to explore the utility of treating sleep apnea (e.g., CPAP in the case of OSA) or treating the underlying medical conditions (e.g., heart failure in central sleep apnea), and the treatment of Cushing’s syndrome could have any influence on the prevalence of these diseases.

## Conclusions

The morbidity and mortality of untreated sleep apnea are well known and could have a slightly higher negative impact on the outcomes in subpopulation groups such as those with Cushing’s syndrome. Oftentimes, as clinicians, we have tunnel vision and overlook underlying coexisting medical conditions, especially disorders such as obstructive sleep apnea. OSA is one of the medical disorders that is often missed during diagnosis and is the most common underrecognized and underdiagnosed medical condition. This study sheds light on sleep apnea and the importance of screening it among patients diagnosed with Cushing’s syndrome. This study also helps bring awareness regarding the possibility of an association between Cushing’s syndrome and sleep apnea among physicians in different fields of practice, including internal medicine, family medicine, sleep medicine, endocrine, and neurology, while caring for patients in their respective areas of practice.
